# Neutrophil-derived S100A8/A9 promotes apoptosis of intestinal epithelial cells in children with duodenal ulcers

**DOI:** 10.18632/aging.204842

**Published:** 2023-07-12

**Authors:** Rong Cheng, Xiaowei Xia, Rong Liu, Wenjun Zhang, Juan Du, Maoyan Zhang, Chuanying Li

**Affiliations:** 1 Department of Gastroenterology, Children's Medical Center of Anhui Medical University (Anhui Provincial Children’s Hospital), The Fifth Clinical College, Anhui Medical University, Hefei 230051, Anhui, China; 2 School of Basic Medical Sciences, School of Basic Medical Sciences, Anhui Medical University, Hefei 230032, Anhui, China

**Keywords:** S100A8/S100A9, children with duodenal ulcers, duodenum, apoptosis

## Abstract

Duodenal ulcer significantly reduces quality of life and safety in children; however, the mechanism of the pathogenesis in children with duodenal ulcer remains unclear. S100A8/A9, which plays a critical role in the occurrence and development of inflammation, has attracted a lot of interest recently. Here, we identified that S100A8/A9 are highly expressed in the serum of children with duodenal ulcers, and this is of excellent diagnostic value. Animal experiments have proved that inhibition of S100A8/A9 can repair ulcer progression. In addition, further study has shown that S100A8/A9, mainly produced by neutrophil, can enhance the apoptosis of intestinal epithelial cells and promote the growth in children with duodenal ulcers. Thus, our research proves the value of S100A8/A9 in the diagnosis and treatment of children with duodenal ulcers.

## INTRODUCTION

*Helicobacter pylori* (*H. pylori*) is the most common infection among the world’s population [1]. According to statistics, about 50% of the world’s population has been notified of *H. pylori* infection, and it is mainly obtained in early childhood [2]. Studies have indicated that *H. pylori* is a major reason of duodenal ulcers and chronic gastritis in children and the high prevalence of *H. pylori* infection in children with duodenal ulcers compared with children with gastric ulcers (range, 33 to 100%; median, 92%) [3, 4]. In addition, genetics, improper diet, and stress are also important causes of duodenal ulcers. Thus, finding an effective method for diagnosing and treating duodenal ulcers is important.

S100A8/A9 were firstly found in the synovial fluid of rheumatoid arthritis (RA) patients [5]. Nowadays, S100A8/A9 are considered to be risk factors for many inflammatory diseases such as inflammatory bowel disease (IBD) [6–8]. Studies show that, S100A8/A9 were largely secreted by neutrophils and macrophages and induce more cytokines to be released by macrophages and exacerbate the progression of inflammatory disease [9, 10]. However, this role of S100A8/A9 in children with duodenal ulcers is still unclear.

In the current study, we examined the expression of S100A8/A9 in the serum of children with duodenal ulcers and also investigated the apoptotic effect of S100A8/A9 on intestinal epithelial cells.

## MATERIALS AND METHODS

### Clinical samples

The children who have recurrent abdominal pain and other gastrointestinal symptoms (vomiting, nausea, loose stools, and constipation) were scheduled for a gastroduodenoscopy. 50 cases specimens with the above symptoms and mucosal edema of the duodenal bulb seen microscopically (no inflammation by pathological examination) were used as Control group. 50 cases specimens of duodenal bulb ulcers were seen endoscopically (with *H. pylori* infection confirmed by rapid urease test) as *H. pylori*^+^DU^+^ group. 20 cases specimens of duodenal bulb ulcers were seen endoscopically (without *H. pylori* infection confirmed by rapid urease test) as *H. pylori*^−^DU^+^ group. 20 cases specimens with the above symptoms and mucosal edema of the duodenal bulb were seen microscopically (with *H. pylori* infection confirmed by rapid urease test) as *H. pylori*^+^DU^−^ group. Demographic and clinical characteristics of patients were listed in Table 1. This study was approved by the Ethics Committee of the Anhui Provincial Children’s Hospital. Informed consent to participate was obtained from children’s parents.

**Table 1:** Demographic and clinical characteristics of patients with Control, *H. pylori*^+^DU^+^, *H. pylori*^−^DU^+^ or *H. pylori*^+^DU^−^ patients who provided serum samples.

**Parameter**	**Control (*n* = 50)**	***H. pylori*^+^DU^+^ (*n* = 50)**	***H. pylori*^−^DU^+^ (*n* = 20)**	***H. pylori*^+^DU^−^ (*n* = 20)**
	**Mean ± standard error**			
Age, mean ± s (y)	8.91 ± 2.9	11.25 ± 2.1	10.02 ± 3.0	6.89 ± 1.93
Weight (kg)	23.1 ± 5.24	34.13 ± 10.15	30.58 ± 6.42	20.50 ± 3.52
Male sex (%)	50	46	55	60
*H. pylori*^+^ (%)	0	100	0	100
DU (%)	0	100	100	0
White blood cell count	7.15 ± 1.59	10.15 ± 6.35	9.87 ± 5.59	15.69 ± 3.25^$^
Neutrophils (%)	39.58 ± 8.91	64.75 ± 20.02^*^	56.57 ± 18.25^#^	40.99 ± 11.35
Lymphocytes (%)	49.74 ± 8.95	30.21 ± 15.43^*^	35.01 ± 16.72^#^	45.06 ± 10.07
Monocytes (%)	6.54 ± 1.20	6.35 ± 2.67	6.11 ± 2.40	6.25 ± 4.60
Eosinophils (%)	3.48 ± 1.98	2.79 ± 2.22	2.89 ± 1.91	3.05 ± 1.44
Basophils (%)	0.41 ± 0.18	0.50 ± 0.72	0.55 ± 0.62	0.39 ± 0.50
Hemoglobin (g/L)	130.03 ± 8.13	121.44 ± 8.16	120.98 ± 10.13	135.57 ± 30.12
Platelets (10^9^/L)	279.18 ± 75.31	319.98 ± 80.64	307.85 ± 13.73	286.55 ± 79.83

^*^P < 0.05, *H. pylori*^+^DU^+^ vs. Control, ^#^*P* < 0.05, *H. pylori*^−^DU^+^ vs. Control, ^$^*P* < 0.05, *H. pylori*^+^DU^−^ vs. Control.

### Animals

Male C57BL/6 J mice (age, 5 weeks; weight, 20–25 g) were obtained at Shanghai SLAC Laboratory Animal Co., Ltd. (Shanghai, China). Mice were kept in a well-ventilated rearing room with a 12-hour light-dark cycle and an ambient temperature of 23 ± 2°C and 70% humidity. They had freedom of choice to obtain water and food. All mice studies were permitted by the Animal Care Committee of Anhui Medical University (Hefei, China).

### Experimental design

The mice were separated in 2 groups with 9 mice in each group. In the first group, the mice were given normal water, the second group mice were given cysteamine (450 mg/kg PO) at 8 AM and 12 PM [11]. After 24 h, the S100A8/A9 inhibitor Paquinimod (5 mg/kg/day) was instilled by caudal vein injection, three mice in each group, and three others with solvent. After 24 h, all mice had been euthanized under ether anesthesia and the duodena carefully removed.

### HE staining

Dewaxing and hydration of the sections were done. The slices were subsequently stained with haematoxylin solution for 5 min under room temperature. Following fractionation by 1% acid alcohol with 1 min, the slices were hatched in 1% eosin with 15 s.

### TUNEL

NCM460 cells were treated with paraformaldehyde solution (4%) at room temperature and fixed for 25 min. After washing three times with phosphate-buffered saline (PBS), the cells were permeabilized by incubating the samples with 0.1% Triton X-100 at room temperature for 5 min. Once permeabilization was done, the samples were washed with PBS three times and incubated in balanced buffer at room temperature for 30 min. Next, the appropriate amount of BrightGreen label mixture was added according to the instructions and incubated for 1 hour at 37°C. Nuclei were stained with DAPI (4′,6-diamidino-2-phenylindole). TUNEL assay results in cell images are captured by fluorescence microscopy (Olympus, Japan), and the results were analyzed by ImageJ software (National Institutes of Health, Bethesda, Maryland).

### Neutrophil isolation

Neutrophils were isolated from peripheral blood in children with duodenal ulcer. Our steps mainly follow the instructions of the peripheral blood neutrophil isolation kit (TBD; Tianjin, China). The separated solution was added in fresh blood and centrifuged at 500 × g for 25 min in 4°C. After centrifugation, removed the neutrophil layer in a new centrifuge tube and added washing solution for three times. Finally, neutrophils were cultured in 24-well plates with 1 × 10^5^ cells in each well using RPMI 1640 medium.

### ELISA analysis

The levels of S100A8 and S100A9 were detected in serum using a commercial ELISA kit (Elabscience, Wuhan, China) based on the directions of the product.

### Data availability

The data used to support the findings of this study are available from the corresponding author upon request.

## RESULTS

### S100A8/A9 were significantly elevated in the serum of children with duodenal ulcer and have diagnostic potential

**Figure 1:**
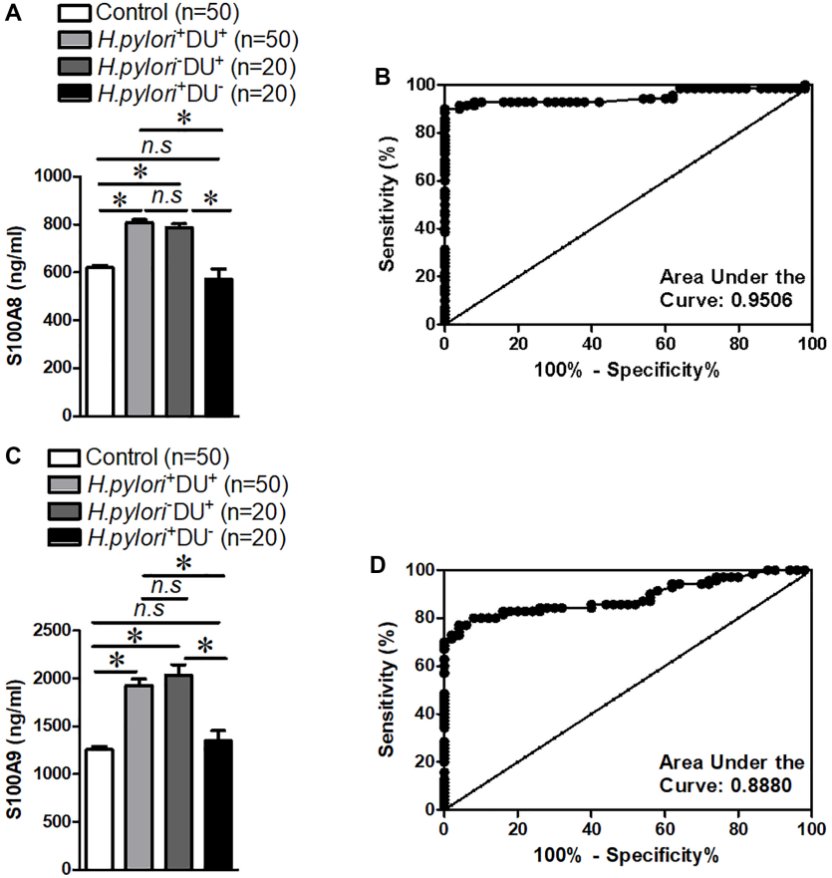
**S100A8/A9 expression and receiver operating characteristic curve analyses in the serum of children with duodenal ulcer.** (**A**) Summary of S100A8 expression. (**B**) Receiver operating characteristic curve analyses of S100A8. (**C**) Summary of S100A9 expression. (**D**) Receiver operating characteristic curve analyses of S100A9. *H.pylori*^−^: non-*Helicobacter pylori* infected, *H.pylori*^+^: *Helicobacter pylori* infected, DU^−^: non-duodenal ulcer, DU^+^: duodenal ulcer. ^*^P < 0.05 compared with Control or *H.pylori*^+^DU^+^ group or *H.pylori*^−^DU^+^ group, *n.s* > 0.05 compared with Control or *H.pylori*^+^DU^+^ group.

In order to demonstrate whether S100A8/A9 were differently expressed in children with duodenal ulcer, the serum samples were collected in children with duodenal ulcer and people with non-ulcer, and the S100A8/A9 expression in children with duodenal ulcer was detected by ELISA. These findings indicated a significantly high expression of S100A8/A9 in the serum of children with duodenal ulcer and *H. pylori*^+^ or *H. pylori*^−^ cannot affect the expression of S100A8/A9 (Figure 1A and 1C). Additionally, receiver operating characteristic curve (ROC) analysis demonstrated excellent diagnostic value of S100A8/A9 in children with duodenal ulcers (Figure 1B and 1D). Our data indicated that S100A8/A9 were expressed with high level of diagnostic value in children with duodenal ulcer and S100A8/A9 is probably a diagnostic and therapeutic target for children with duodenal ulcers.

### Inhibition of S100A8/A9 can effectively alleviate intestinal epithelial damage in duodenal ulcer mice

**Figure 2:**
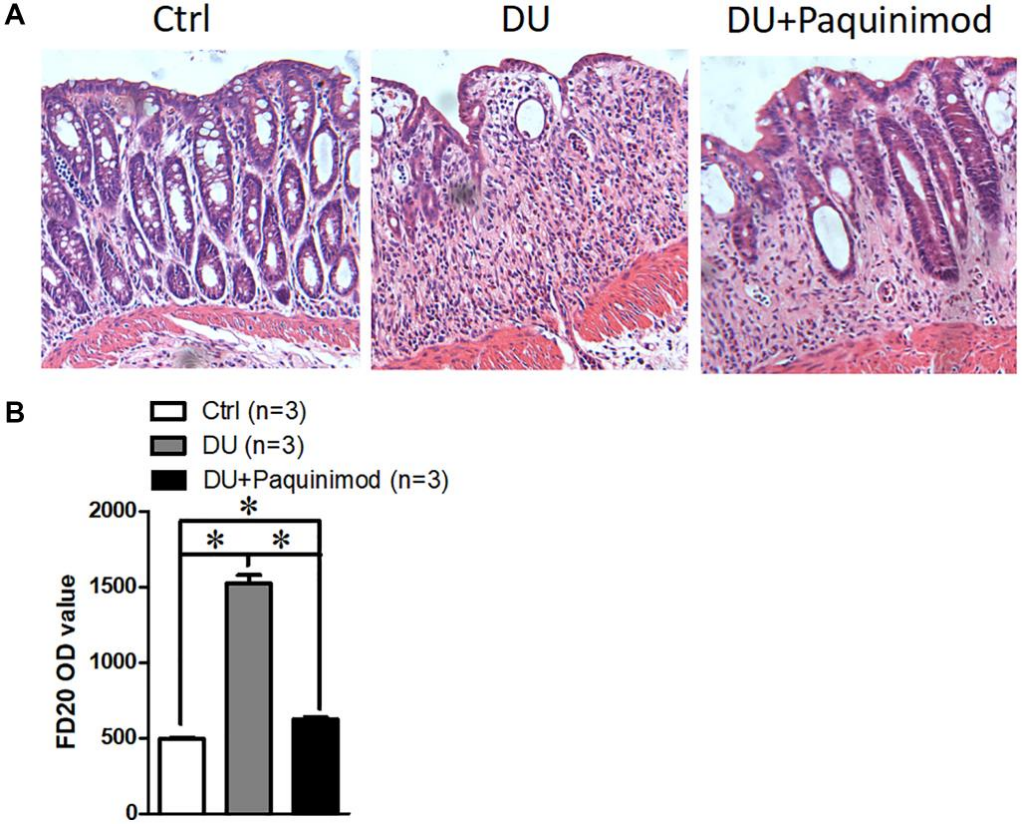
**Suppression of S100A8/A9 relieves the development of duodenal ulcer.** (**A**) The representative staining images show hematoxylin-eosin staining of duodenal tissue. (**B**) FD20 content in serum. Paquinimod: S100A8/A9 inhibitor. ^*^P < 0.05 compared with Ctrl.

To clarify the role of S100A8/A9 in duodenal ulcer, we prepared mice with duodenal ulcer and then administered S100A8/A9 inhibitors-Paquinimod [12], and HE results showed that the degree of damage to the intestinal epithelium was significantly restored (Figure 2A). Intestinal epithelial barrier is a major factor in inflammatory bowel disease [13]. Thus, we also examined the change in the permeability of the intestinal epithelium after treating with Paquinimod in duodenal ulcer disease. The results suggested that S100A8/A9 inhibitor Paquinimod can significantly reduce intestinal epithelial permeability in mice with duodenal ulcer (Figure 2B). These results reminded us that intestinal epithelial damage in duodenal ulcers may be caused by increased S100A8/A9.

### S100A8/A9 dramatically contributed to the apoptosis of NCM460 cells

**Figure 3:**
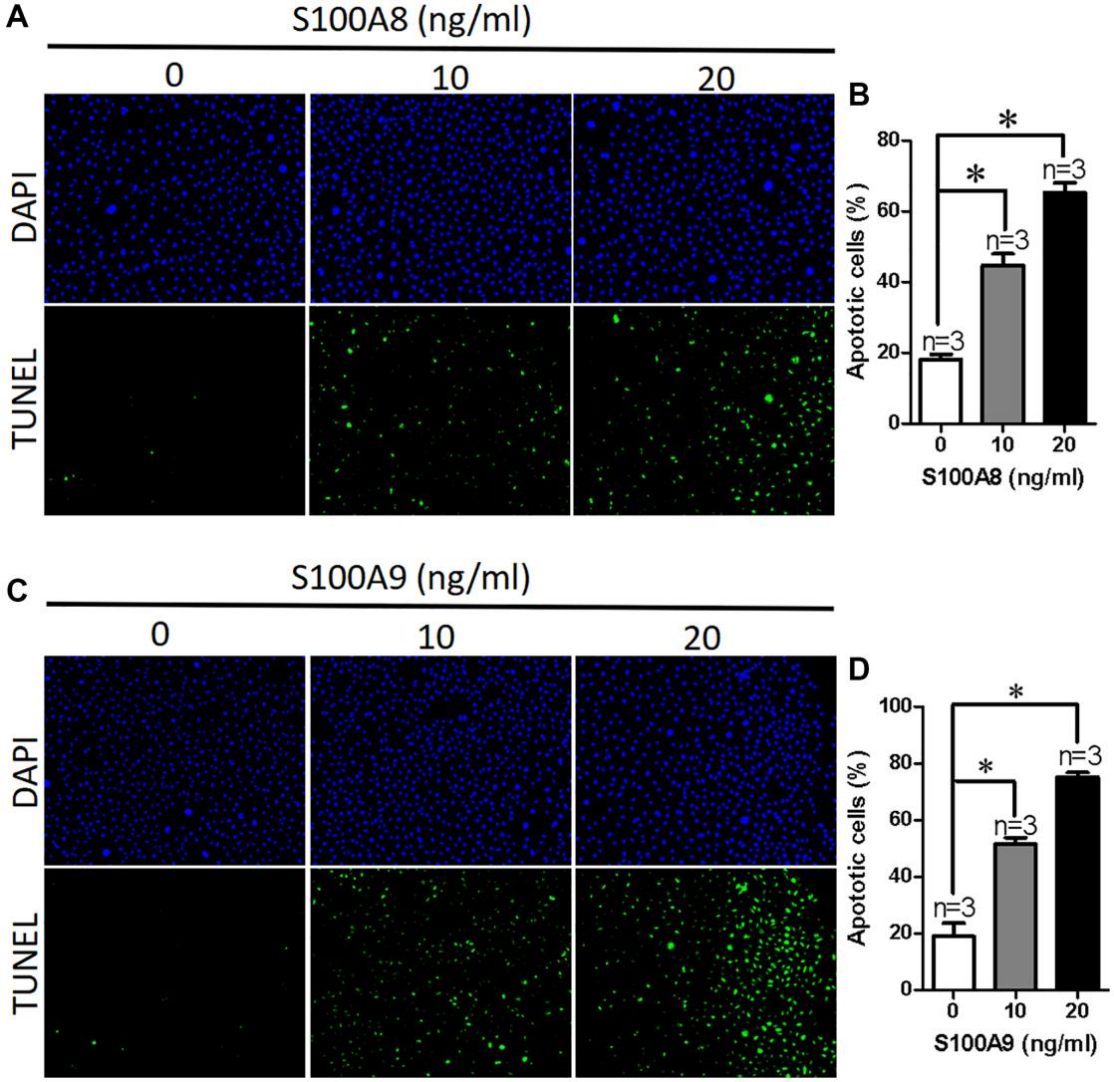
**S100A8/A9 promotes apoptosis in NCM460 cells.** (**A**) Representative images showing DAPI and TUNEL in NCM460 cells treated with S100A8 (0, 10, 20 ng/ml). (**B**) Summary data showing the percentage of apoptotic NCM460 cells after treated with S100A8. (**C**) Representative images showing DAPI and TUNEL in NCM460 cells treated with S100A9 (0, 10, 20 ng/ml). (**D**) Summary data showing the percentage of apoptotic NCM460 cells after treated with S100A9. DAPI: blue; 4′,6-diamidino-2-phenylindole. TUNEL: green; terminal deoxynucleotidyl transferase dUTP nick end labeling. ^*^P < 0.05 compared with 0 ng/ml.

To investigate how high expression of S100A8/A9 leads to damage of the intestinal epithelium, we used different concentrations of S100A8 or S100A9 proteins to treat NCM460 cells for 24 hours, and then used TUNEL assay to determine the apoptosis of NCM460 cells. Our findings showed that the apoptotic cell percentage increased in a concentration-dependent manner for S100A8 (Figure 3A and 3B). We then treated NCM460 cells with S100A9 protein and found a similar effect, increasing the percentage of apoptotic cells in a concentration-dependent manner (Figure 3C and 3D). All findings demonstrate that S100A8 and S100A9 may cause intestinal injury by promoting apoptosis of epithelial cells.

### Serum S100A8/A9 in children with duodenal ulcer is mainly derived from neutrophils

**Figure 4:**
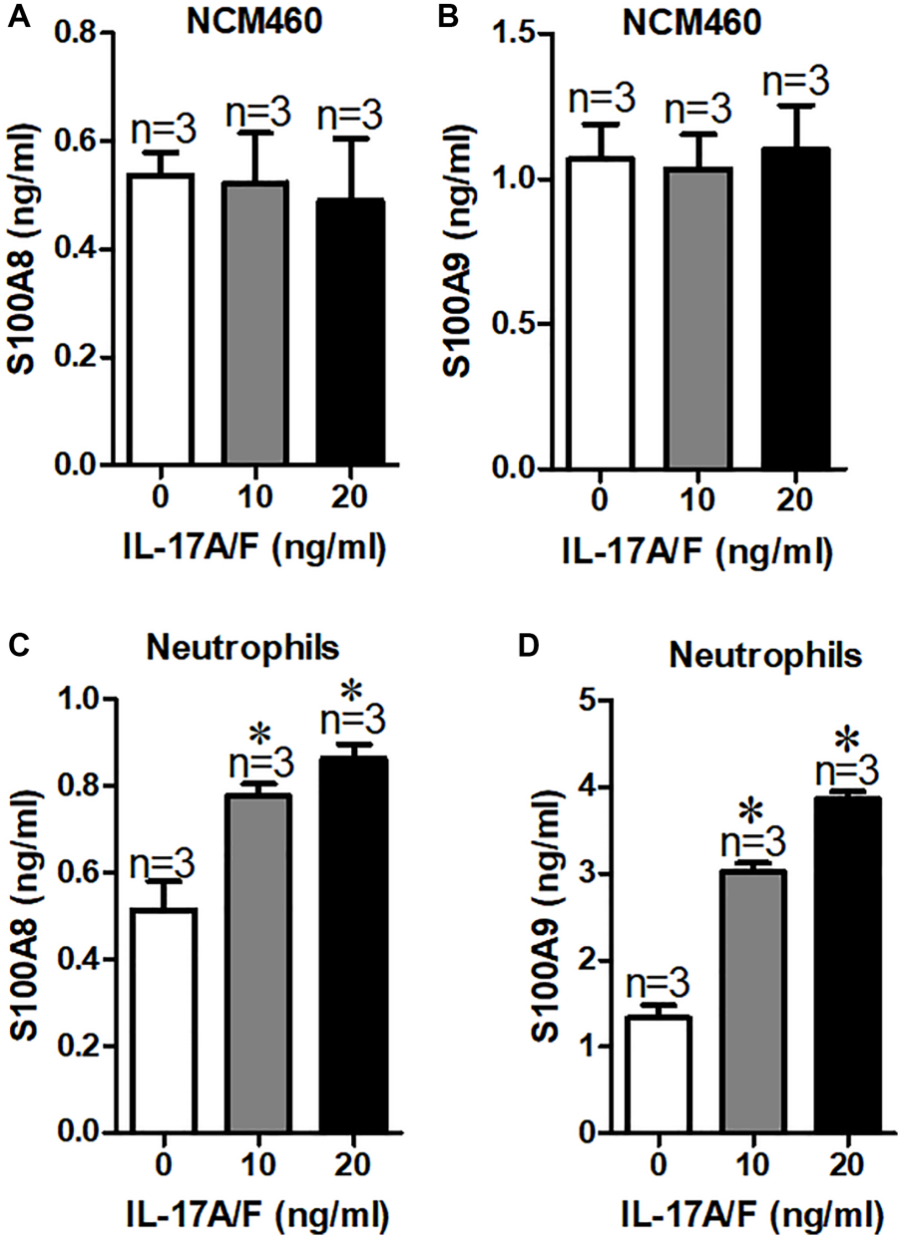
**Serum S100A8/A9 are mainly derived from neutrophils in children with duodenal ulcer.** (**A**, **B**) Summary of serum S100A8 (**A**) and S100A9 (**B**) expression in culture medium of NCM460 cells after treated with IL-17A/F. (**C**, **D**) Summary of serum S100A8 (**C**) and S100A9 (**D**) expression in culture medium of neutrophils cells after treatment with IL-17A/F. ^*^P < 0.05 compared with 0 ng/ml.

To identify the source of elevated the levels of S100A8 and S100A9 in children with duodenal ulcer, here, we analyzed the signaling pathways generated by S100A8 and S100A9, we found that IL-17 signaling pathway is involved in S100A8 and S100A9 production [14]. Therefore, we treated NCM460 cells with different concentrations of IL-17A/F and used ELISAs to detect S100A8 and S100A9 concentrations released into the cell culture medium. Our results showed that the secretion of S100A8 or S100A9 in NCM460 cells was not increased by IL-17A/F (Figure 4A and 4B). To learn more about its origins, we reviewed relevant information and found high levels of S100A8/A9 in neutrophils and monocytes [15]. And Bai et al. suggested that IL-17 can stimulate neutrophils to release S100A8/A9 in mycoplasma pneumonia-induced pneumonia in children [14]. Therefore, we examined the expression of S100A8/A9 in neutrophils from children with duodenal ulcers after stimulation of IL-17A/F. The results indicated a significant increase in the secretion of S100A8/A9 from neutrophils by IL-17A/F (Figure 4C and 4D). Therefore, the elevated levels of S100A8/A9 in the serum of children with duodenal ulcers are probably caused by IL-17-induced reduction of neutrophils.

## DISCUSSION

Duodenal ulcer is a difficult disease to treat that also occurs in children and severely damages the safety of people’s lives and quality of life [16, 17]. In most cases, the diagnosis and the mechanism of children with duodenal ulcers remain unknown [18]. The expression of S100A8/A9 are regulated during inflammation [9]. The effect of S100A8/A9 in children with duodenal ulcers is still unknown. Here, we identified a significant increase in the expression of S100A8/A9 in children with duodenal ulcers and it may contribute to the development of duodenal ulcer via promoting the apoptosis of intestinal epithelium. Major findings presented in this study are as follows: (1) S100A8 and S100A9 were obviously increased in children with duodenal ulcer and had excellent values for the diagnosis of children with duodenal ulcer. (2) Inhibition of S100A8/A9 can effectively alleviate intestinal epithelial damage in duodenal ulcer mice. (3) Apoptosis of NCM460 cells were significantly induced by S100A8/A9 in a concentration-dependent manner. (4) Release of S100A8/A9 by IL-17A/F induced neutrophils, but not NCM460 cells. In a word, we proposed S100A8/A9 as a novel biomarker for the clinical differential diagnosis of children with duodenal ulcer.

*H. pylori* infection has been considered to be a major cause of duodenal ulcer development in children [19]. However, not every case of duodenal ulcers is induced by *H. pylori* infection [20]. Duodenal ulcer is very dangerous and can lead to bleeding and other complications. Thus, exploring its specifically pathogenic mechanisms and finding specific therapeutic targets are important. Nowadays, the main treatment for duodenum is antibacterial therapy [21]. However, due to the heavy use of antibiotics, drug-resistant *H. pylori* gradually appeared. It is critical to find new treatments and treatment targets. In our research, a significant high expression of S100A8/A9 in children with duodenal ulcer was found to be of good diagnostic value. And S100A8/A9 expression is increased in both *H. pylori*^−^ and *H. pylori*^+^ DU patients, which may be due to inflammation, which is also consistent with other inflammatory diseases [14]. This finding could prove the new clues and targets for the diagnosis and treatment of duodenal ulcers in children. However, it may also need to be combined with other symptoms, such as, abdominal pain.

S100A8 and S100A9 are belonging to S100 family and are Ca^2+^ binding proteins [9]. Studies showed that S100A8/A9 play a critical role in the occurrence and development of inflammation [9]. For example, Sreejit et al. reported that S100A8/A9 promote inflammation in myocardial infarction [22]. Wang et al. suggested that circulating levels of S100A8/A9 indicate intraocular inflammation in patients with uveitis [23]. Kang et al. showed the role of S100A8/A9 acting as a biomarker of synovial inflammation and joint injury for patients with rheumatoid arthritis [24]. In the present work, our study suggested that the apoptosis of intestinal epithelial cells was significantly induced by S100A8/A9 in a concentration-dependent manner. Intestinal epithelial cells damage is an essential stage in the progression of intestinal diseases [25]. Furthermore, cell apoptosis is a key mechanism in the defensive loss of epithelial cells [26]. Therefore, S100A8/A9 may be newly found causative factors that were involved in children with duodenal ulcers.

S100A8/A9 has been reported to be mainly produced by neutrophils and macrophages [9]. During inflammatory diseases, neutrophils or macrophages move to areas of inflammation and can produce S100A8/A9, thereby accelerating inflammation [27, 28]. Studies suggested that the expression of S100A8/A9 is promoted by IL-17. For example, Lima et al. found IL-17-producing cells show inflammasome activation and express S100A8/A9 in hidradenitis suppurativa [29]. Bai et al. suggested that S100A8/A9 were highly expressed after IL-17A treatment in children with mycoplasma pneumoniae-induced pneumonia [14]. In our research, IL-17A/F stimulated neutrophil to produce more S100A8/A9 which can promote intestinal epithelial cells apoptosis. Hence, through investigating the potential molecular mechanisms, we identified the causative role and source of S100A8/A9 in children with duodenal ulcers. We prove a perspective on how neutrophils could be potentially targeted to ameliorate the development of children with duodenal ulcers.

## CONCLUSION

In conclusion, our present study indicated that S100A8/A9 are expressed highly in the serum of children with duodenal ulcers. Further studies suggest that S100A8/A9, derived from neutrophil, may deepen the development of duodenal ulcers by promoting apoptosis of intestinal epithelial cells.
